# Contribution of Other Respiratory Viruses During Influenza Epidemic Activity in Catalonia, Spain, 2008–2020

**DOI:** 10.3390/microorganisms12112200

**Published:** 2024-10-31

**Authors:** Nuria Torner, N. Soldevila, L. Basile, M. M. Mosquera, P. de Molina, M. A. Marcos, A. Martínez, M. Jané, A. Domínguez, The Working Group for the Catalan Influenza and Acute Respiratory Infection Sentinel Surveillance Network (PIDIRAC)

**Affiliations:** 1 CIBER Epidemiology and Public Health CIBERESP, Instituto de Salud Carlos III, 28029 Madrid, Spain; nsoldevila@ub.edu (N.S.);; 2 Department of Medicine, University of Barcelona, 08036 Barcelona, Spain; 3 Public Health Agency of Catalonia, 08005 Barcelona, Spain; 4 Department of Microbiology, Hospital Clínic of Barcelona, University of Barcelona, 08036 Barcelona, Spain; mdmosquera@clinic.cat (M.M.M.);; 5 Barcelona Institut of Global Health (ISGLOBAL), 08036 Barcelona, Spain

**Keywords:** influenza, respiratory viruses, seasonal epidemic activity, vaccination, influenza-like illness (ILI)

## Abstract

Background: During seasonal influenza activity, circulation of other respiratory viruses (ORVs) may contribute to the increased disease burden that is attributed to influenza without laboratory confirmation. The objective of this study was to characterize and evaluate the magnitude of this contribution over 12 seasons of influenza using the Acute Respiratory Infection Sentinel Surveillance system in Catalonia (PIDIRAC). Methods: A retrospective descriptive study of isolations from respiratory samples obtained by the sentinel surveillance network of physicians was carried out from 2008 to 2020 in Catalonia, Spain. Information was collected on demographic variables (age, sex), influenza vaccination status, epidemic activity weeks each season, and influenza laboratory confirmation. Results: A total of 12,690 samples were collected, with 46% (5831) collected during peak influenza seasonal epidemic activity. In total, 49.6% of the sampled participants were male and 51.1% were aged <15 years. Of these, 73.7% (4298) of samples were positive for at least one respiratory virus; 79.7% (3425 samples) were positive for the influenza virus (IV), with 3067 samples positive for one IV type, 8 samples showing coinfection with two types of IV, and 350 showing coinfection of IV with more than one virus. The distribution of influenza viruses was 64.2% IVA, 35.2% IVB, and 0.1% IVC. Of the other respiratory viruses identified, there was a high proportion of human rhinovirus (32.3%), followed by human adenovirus (24.3%) and respiratory syncytial virus (18; 7%). Four percent were coinfected with two or more viruses other than influenza. The distribution of coinfections with ORVs and influenza by age groups presents a significant difference in proportions for 0–4, 5–14, 15–64 and >64 (21.5%, 10.8%, 8.2% and 7.6%: *p* < 0.001). A lower ORVs coinfection ratio was observed in the influenza-vaccinated population (11.9% vs. 17.4% OR: 0.64 IC 95% 0.36–1.14). Conclusions: During the weeks of seasonal influenza epidemic activity, other respiratory viruses contribute substantially, either individually or through the coinfection of two or more viruses, to the morbidity attributed to influenza viruses as influenza-like illness (ILI). The contribution of these viruses is especially significant in the pediatric and elderly population. Identifying the epidemiology of most clinically relevant respiratory viruses will aid the development of models of infection and allow for the development of targeted treatments, particularly for populations most vulnerable to respiratory viruses-induced diseases.

## 1. Introduction

Seasonal influenza viruses are responsible for a huge number of respiratory tract infections every year [1] and the implementation of sentinel surveillance programs as well as the development of tools such as mathematical models of influenza virus transmission have been key to understanding its epidemiology, thus enabling public health planning for preparedness and control.

Influenza A (IVA) and B (IVB) viruses cause seasonal epidemics of influenza disease, yet IVA also has pandemic potential as its reservoir of infection is not only humans, but also animal species. According to their two surface proteins, hemagglutinin (HA) and neuraminidase (NA), which are responsible for the infectious cycle of the influenza virus, IVA is further classified into subtypes according to HA and NA combinations. Currently circulating in humans are subtypes A(H1N1)pdm09 and A(H3N2). Influenza B viruses are classified into lineages: the B/Yamagata or the B/Victoria lineage [2]. However, recent evidence suggests that the B/Yamagata lineage is no longer circulating. Soon after the start of the COVID-19 pandemic, the detection of influenza B/Yamagata cases decreased globally. The likelihood of extinction implies a response in terms of reassessing the composition of influenza vaccines and a possible change in the biosafety level when handling B/Yamagata viruses in laboratories [3].

Influenza viruses often circulate during colder months and can lead to significant illness, especially in vulnerable populations. Coinfection with other respiratory viruses can complicate diagnosis, severity, and treatment, particularly during peak epidemic seasons.

There is increasing evidence that microbes do not always cause disease individually but can act in combination with other pathogens. In the case of influenza viruses, this is also the case and may lead to erroneous interpretation of transmission patterns and severity [4]. Many pathogens, bacteria or viruses can interact with influenza, and it is important to be able to assess the magnitude of these interactions. Interactions between viruses and bacteria in acute respiratory infections have been reported, especially the synergism between influenza virus and *S. pneumoniae* [5,6,7], yet the coexistence of viral pathogens is not well documented.

If bacterial/viral coinfections appear to be linked to more severe clinical presentations, this might not always be the case when looking at viral coinfections. A review from Esneau et al. [8] concluded that milder RV infection may stimulate innate antiviral immunity in the upper respiratory tract and thus could have a protective effect against influenza and SARS-CoV-2. The importance of understanding the dynamics of respiratory viral coinfections lies in their potential and ability to worsen disease outcomes, and it seems clear that respiratory coinfection is now more common than was previously thought [9].

The availability of accurate multiplex panels for viral pathogen detection implemented in sentinel surveillance allows for information about circulating respiratory viruses to be obtained, as well as correct clinical management of respiratory tract infections, without leading to unnecessary antibiotic/antiviral treatments [10]. Infections due to influenza viruses as well as ORVs such as respiratory syncytial virus (RSV), parainfluenza virus (PIV), human metapneumovirus (HMPV), enterovirus, human rhinovirus (HRV), adenovirus (ADV), and human coronavirus (HCoV) account for a significant proportion of acute respiratory tract infections. However, acute respiratory tract infections (ARIs) of viral etiology have similar clinical presentations, making it, to say the least, difficult to distinguish between etiologic agents. Nonetheless, differentiating between infections caused by these viruses may help policy makers to make decisions related to vaccination and resource allocations [11].

There are several studies focusing on the viral etiology of acute respiratory infection (ARI) in children and the importance of some viruses, such as influenza and respiratory syncytial virus (RSV) [12]. There even are some studies on the disease burden and hospital admission among the elderly population, which rank it similarly to that of seasonal influenza A virus infection in some seasons [13,14], yet the burden of disease caused by ORVs in primary care and for all ages has not been well described.

The understanding of competence of influenza and other respiratory viruses is also important for the public health assessment of influenza preventive measures. In Catalonia, the sentinel surveillance system (PIDIRAC) set up in 1998 monitored influenza viruses as well as other respiratory viruses, such as respiratory syncytial virus (RSV), rhinovirus, adenovirus, or coronaviruses. From 2008 onwards, the representativeness of the sentinel system and availability of virological data improved. The sentinel surveillance in the primary care network represented approximately 1% of the entire population of Catalonia, being distributed geographically in a representative manner according to the population density of the area. Sampling was carried out by each sentinel physician upon consultations with influenza-like illness symptoms (60 physicians, encompassing pediatricians and general practitioners). Around 1000 samples were collected per season, with a higher rate of sampling during epidemic weeks. By the end of the 2019–2020 season, with the irruption of SARS-CoV-2, the system had been redesigned to cope with new needs for surveillance evidenced by the COVID-19 pandemic. This work is a compilation of all virological data provided by the PIDIRAC primary care sentinel surveillance system during its last 12 seasons of activity as such. This study aims to describe the epidemiology of confirmed respiratory virus infections during 12 influenza seasons in Catalonia to assess the effect that other than influenza respiratory viruses (ORVs) have on the influenza-like illness (ILI) activity reported.

## 2. Material and Methods

### 2.1. Study Design

The study conducted was a retrospective study of the etiology and epidemiological characteristics of cases of acute respiratory tract infection (ARI) and influenza-like illness (ILI) attended by the primary care influenza sentinel physicians’ network (PIDIRAC) during twelve influenza seasons (from 2008–2009 to 2019–2020) in Catalonia. This region in the northeast of Spain has a population of approximately 7.6 million inhabitants and the PIDIRAC sentinel network covers approximately 1% of the total population homogenously distributed throughout the territory in a proportional manner according to population density.

Each sentinel physician collected weekly nasopharyngeal swabs in up to two individuals who presented symptoms compatible with ARI or ILI and sent them to the Influenza Viruses Reference Laboratory at Hospital Clinic of Barcelona for determination of a panel of respiratory viruses [IV A-C, RSV, PIV 1-4, ADV, HCoV, HRV, HMPV and bocavirus (HBV)] by real-time reverse transcriptase-polymerase chain reaction (RT-PCR) [15]. Viral coinfection was defined as the simultaneous identification of two or more viruses in a single respiratory sample.

Sample data were distributed into 4 age groups (0–4, 5–14, 15–64 and >64 years) according to the standardized criteria set up for the surveillance system.

### 2.2. Data Collection

For each individual, the following variables were considered: age; sex; comorbidities, such as chronic cardiovascular disease (CVD), chronic obstructive pulmonary disease (COPD), asthma, liver and kidney disease, diabetes mellitus (DM), obesity [defined as a body mass index (BMI) > 40 kg/m^2^ for adults and a BMI at or above the 95th percentile for children and teens of the same age and sex]; and immunodeficiency and virology laboratory results. The term “comorbidity” has been used to refer to any long-term health condition that coexists in an individual with a specific condition of interest, in this case ILI [16].

A temperature of ≥37.8 °C was used to assess the WHO ILI case definition, as this is the variable recorded on the form used by sentinel physicians. Influenza epidemic weeks were defined each season according to the data provided by the PIDIRAC sentinel surveillance system’s final seasonal reports.

### 2.3. Statistical Analysis

Proportions between the types of detected virus (influenza and/or ORVs) and between the age groups (0–4, 5–14, 15–64 and >64 y.o.) were compared using the χ2 test or Fisher’s exact test, where appropriate. We estimated odds ratio (OR) and 95% confidence intervals (CIs) using logistic regression models, comparing distribution of cases in which the influenza virus was detected with cases positive for other respiratory viruses or in coinfection with or without the influenza virus. The association between these results and influenza vaccination status was assessed. We also estimated the association of ORV infection compared with influenza virus infection with the different comorbidities collected by calculating ORs and their 95% CIs using logistic regression models. Multivariate logistic regression was performed, adjusting by age and sex. These calculations were made for twelve influenza epidemic seasons, considering the period from the first to the last epidemic week according to the surveillance system information reports per season.

The analysis was performed using the SPSS version 25 statistical package and R version 3.5.0 statistical software.

### 2.4. Ethical Considerations

All data were collected as part of routine public health surveillance activities according to the legal mandate of the Health Department of Catalonia, which is officially authorized to receive, treat and temporarily store personal data in cases of infectious diseases [17]. All data were fully anonymized. All study activities were part of public health surveillance and were exempt from institutional board review and did not require informed consent.

## 3. Results

**Figure 1:**
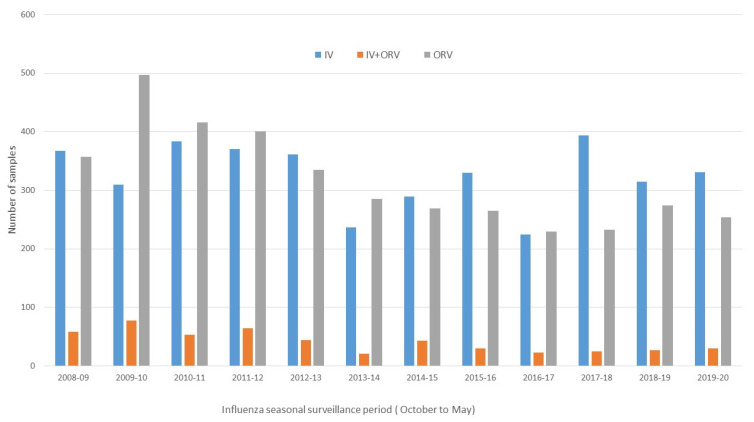
Distribution of influenza-like illness samples collected by the PIDIRAC primary care influenza surveillance of Catalonia during surveillance periods (from October to May) within each season. PIDIRAC 2008–2020.

**Figure 2:**
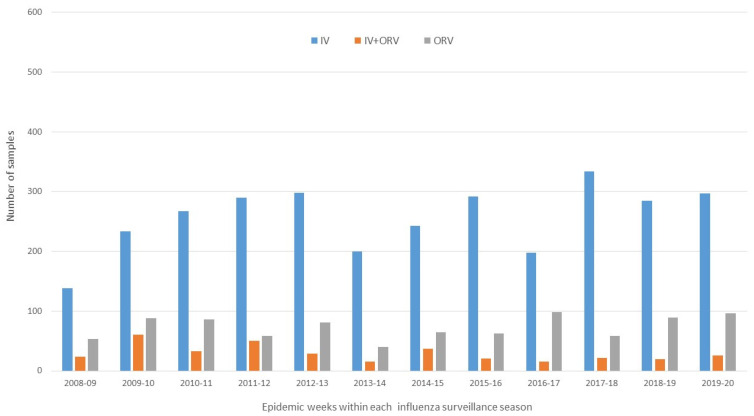
Distribution of influenza-like illness samples collected by the PIDIRAC primary care influenza surveillance of Catalonia during epidemic activity weeks within each season. PIDIRAC 2008–2020.

A total of 12,690 samples were collected during the 12 seasons included in the study, with a mean of 1038.5 samples per season (SD ± 213.8), ranging from 772 to 1479 (the highest corresponded to the 2009–2010 pandemic season). Figure 1 and Figure 2 show the distribution of the virological assessments of all ILI samples during the entire influenza seasonal surveillance period (from October to May) and those restrained to epidemic activity weeks within each season.

The distribution of samples according to sex and age showed that 49.6% were male and 51.1% were <15 years. Table 1 shows the distribution of positivity and coinfections out of the entire study sample and within positive samples according to age group. The highest percentage of positivity for IV was observed in the 5–14 years age group (63.5%), while the highest rate of coinfection corresponded to the 0–4 years group (17.6%). The distribution of these proportions among samples positive for any respiratory virus holds the same relation, being 81% for the 5–14 years age group and 21.5% for the 0–4 years age group. Samples collected during influenza seasonal epidemic activity accounted for 46% (5831) of samples. Of these, 73.7% (4298) were positive for at least one respiratory virus, 79.7% (3425) for IV, 3067 for one IV type, 66.2% for IVA, 35.2% for IVB, 0.1% for IVC, 8 for coinfection with two types of IV, and 350 for coinfection of IV with other respiratory viruses (ORVs); 20.3% (873) were positive for ORV, and of these 16.6% (145) were coinfections. The association of positivity for ORVs according to age group showed statistically significant differences among age groups, the most frequent being the 0–4 year age group (OR (IC 95%) 4.85 (3.80–6.18)) (Table 2).

Of other respiratory viruses identified, there was a high proportion of human rhinovirus (32.3%) followed by human adenovirus (24.3%), and respiratory syncytial virus (18; 7%). Four percent of cases presented coinfection with two or more respiratory viruses other than influenza. The distribution of coinfection with ORVs and influenza by age groups presents a significant difference in proportions for 0–4, 5–14, 15–64 and >64 years (21.5%, 10.9%, 8.2% and 7.3%, respectively; *p* < 0.001). A lower coinfection proportion was observed in the influenza-vaccinated population (11.9% vs. 17.4%; OR (IC 95%) 0.64 (0.36–1.14); *p* = 0.13) (Table 3). Among comorbidities, metabolic diseases and immunodeficiency were significantly associated with ORV infection in comparison to influenza virus infection (aOR: 0.44, 95% CI: 0.27–0.73 and aOR: 0.38, 95% CI: 0.17–0.85, respectively) (Table 4).

## 4. Discussion

This study presents a descriptive analysis of the etiology of acute respiratory infection (ARI) in a primary care setting from 2008 to 2020. The information granted by the PIDIRAC Sentinel Surveillance Program of Catalonia has been extremely useful to learn, monitor and manage ARI prior to the onset of the COVID-19 pandemic. Although it has since been replaced by the Sistema d’Informació per a la Vigilància d’Infeccions a Catalunya (SIVIC) in line with the Spanish Sistema de Vigilancia de Infección Respiratoria Aguda (SiVIRA) [18], the huge number of samples collected during these 12 seasons allowed for a detailed assessment of the differences in the distribution of IV as well as ORV that cause great morbidity during winter epidemics and present as influenza-like syndromes.

In this study, the most frequent virus in coinfection with IV was HRV, a fact also observed by Esneau et al. in a review exploring the complex relationship between HRV and disease, concluding that HRVs coexist with many other respiratory pathogens [8]. The IV-RSV coinfection rate found in our study was lower than for HRV or even ADV, and this could be due to viral competition when it comes to RSV and influenza—when RSV infection rates are high, influenza rates of infection are low and the converse is also true [19,20].

On the other hand, Bermúdez et al. observed a high prevalence of viral coinfections (39.2%) among children hospitalized with acute bronchiolitis. The most common virus found in coinfection was RSV. While no statistically significant differences were observed as to sex in our study, the study by Bermúdez et al. did find that viral coinfections were more frequent in girls [21].

The fact that there was a lower coinfection proportion in the influenza-vaccinated population might be due to the induction of trained immunity through influenza vaccination. It has been hypothesized that trained immunity might be an important mechanism underlying these beneficial heterologous effects of vaccines. In this sense, Netea et al. presented suggestive evidence that trained immunity is a fundamental characteristic of host defense of multicellular organisms, including mammals [22]. Debisarun et al. also investigated the induction of trained immunity and the impact on systemic inflammation caused by the influenza vaccine and COVID-19 incidence and found evidence of a protective effect [23].

The fact that comorbidities such as metabolic diseases and immunodeficiency were significantly associated with ORV infection in comparison to influenza virus infection could be attributed to the seasonal influenza recommendation for these risk group populations. This assumption is in line with the observations of Lim et al. stating that immunization uptake could be one of the causes of significant differences between children with and without comorbidities [24].

Among the strengths of this study, we emphasize that the molecular techniques used proved to be highly sensitive in detecting a wide range of respiratory viruses. In addition, the period studied was extensive, which allowed for the collection of a sample of patients during different epidemic seasons, encompassing diverse viral circulation throughout the years. We restricted the study period to seasons before the onset of the pandemic to avoid the substantial variation and reduction in respiratory virus circulation observed during the COVID-19 pandemic [25]. Although the changes in circulation of non-SARS-CoV-2 respiratory viruses cannot be attributed to any one factor, implementation of non-pharmaceutical measures was probably important to reduce community circulation of non-SARS-CoV-2 respiratory viruses [26].

Our study had some limitations. Only primary care samples were included in the analysis and thus comparative severity and burden of disease in viral coinfection as a whole could not be analyzed. Despite the large sample size available in our study, these were obtained by sentinel data which are not exhaustive, and therefore, population incidence rates could not be calculated. Nevertheless, they are a robust proxy to influenza and ORV activity in Catalonia.

Looking at viral–viral coinfections, there is still a lot to learn about respiratory illness coinfections, as the interplay is not yet fully understood. Overall, being able to better understand coinfection relationships can aid in the development of therapeutic methods as well as potentially affect the role of molecular testing in everyday practice [27]. The multiple pathogens in co-circulation can lead to competitive or cooperative forms of interactions. [28]. The potential for coinfections with other seasonal pathogens, and altered baseline host resistance due to vaccination, mean that patterns of disease will continue to change. The significant effect of comorbidity on disease outcome is likely to persist and should remain a research priority [16].

## 5. Conclusions

Future studies should be carried out to help understand viral interactions, replication kinetics, and immune response in coinfections thus providing insights into respiratory viruses’ infection patterns when they coinfect a susceptible host [27].

Improved understanding of how the epidemiology of viral infections is interlinked can help to improve disease forecasting and evaluation of disease control intervention. The study was carried out with data collected before the onset of the COVID-19 pandemic, and thus allows for future comparisons as to the behavior of respiratory viruses before and after the irruption of SARS-CoV-2.

## Figures and Tables

**Table 1:** Distribution of positivity and coinfections according to age group. A. Percentage is related to the total number of samples collected. B. Percentage is related to the total number of positive samples.

A	0–4 years	5–14 years	15–64 years	<64 years
Positivity	82.2%	78.4%	65.9%	63.9%
At least one IV (IV or IV coinfection)	42.9%	63.5%	50.6%	43.6%
At least one ORV (ORV or coinfection of ORV)	28.5%	7.8%	11.2%	16.8%
Coinfection IV + ORV	10.7%	7.2%	4.0%	3.4%
Coinfection ORVs	6.9%	0.9%	1.2%	1.4%
% Coinfection *	17.6%	8.5%	5.3%	4.8%
B	0–4 years	5–14 years	15–64 years	<64 years
At least one IV (IV or IV coinfection)	52.2%	81.0%	76.9%	68.3%
At least one ORV (ORV or coinfection of ORV)	34.8%	9.9%	17.0%	26.4%
Coinfection IV + ORV	13.0%	9.2%	6.1%	5.4%
Coinfection ORVs	8.5%	1.1%	1.9%	2.2%
% Coinfection *	21.5%	10.8%	8.2%	7.6%

* coinfection IV + IV, IV + ORV, ORVs.

**Table 2:** Distribution of positivity for ORVs according to age group.

	0–4 years	5–14 years	15–64 years	<64 years
Positive samples	875	1081	1344	186
ORVs (with or without coinfection)	304 (34.7%)	107 (9.9%)	228 (17.0%)	49 (26.3%)
OR (95% CI)	4.85 (3.80–6.18)	Ref. 1	1.86 (1.45–2.38)	3.26 (2.22–4.77)

**Table 3:** Distribution of positivity for ORVs (A) and IV (B) and coinfections according to vaccination status.

A. Other Respiratory Viruses (ORVs)	Vaccinated	Unvaccinated
Coinfection ORVs	15/126 (11.9%)	13/747 (17.4%)
ORVs without coinfection	111/126 (88.1%)	617/747 (82.6%)
	OR (IC 95%) 0.64 (0.36–1.14); *p* = 0.13	
B. Influenza (IV)	Vaccinated	Unvaccinated
Coinfection IV + ORVs	32/262 (12.2%)	317/3157 (10.0%)
Influenza without coinfection	230/262 (87.8%)	2840/3157 (90.0%)
	OR (IC 95%) 1.25 (0.85–1.84); *p* = 0.26	

**Table 4:** Comorbidities associated with Other respiratiory viruses (ORVs) vs. influenza virus.

	Influenza (N = 3075)	ORV (N = 873)	Crude OR (95% CI)	*p* Value	Adjusted OR (95% CI)	*p* Value
Cardiovascular disease	63 (2.0%)	23 (2.6%)	0.77 (0.48–1.25)	0.30	0.64 (0.39–1.05)	0.08
Chronic respiratory disease	157 (5.1%)	54 (6.2%)	0.82 (0.59–1.12)	0.21	0.86 (0.58–1.28)	0.46
Chronic liver disease	12 (0.4%)	3 (0.3%)	1.14 (0.32–4.04)	0.84	0.82 (0.21–3.11)	0.77
Chronic kidney disease	18 (0.6%)	4 (0.5%)	1.28 (0.43–3.79)	0.66	1.69 (0.48–5.98)	0.42
Metabolic disease	47 (1.5%)	25 (2.9%)	0.53 (0.32–0.86)	0.01	0.44 (0.27–0.73)	0.01 *
Obesity	32 (1.0%)	11 (1.3%)	0.82 (0.41–1.64)	0.58	0.80 (0.33–1.92)	0.62
Immunodeficiency	19 (0.6%)	14 (1.6%)	0.38 (0.19–0.76)	0.01	0.38 (0.17–0.85)	0.02 *

* Statistically significant *p* values in bold.

## Data Availability

The data that support the findings of this study are available from the corresponding author upon reasonable request.
